# Non-Lytic Antibacterial Peptides That Translocate Through Bacterial Membranes to Act on Intracellular Targets

**DOI:** 10.3390/ijms20194877

**Published:** 2019-10-01

**Authors:** Marlon H. Cardoso, Beatriz T. Meneguetti, Bruna O. Costa, Danieli F. Buccini, Karen G. N. Oshiro, Sergio L. E. Preza, Cristiano M. E. Carvalho, Ludovico Migliolo, Octávio L. Franco

**Affiliations:** 1 S-inova Biotech, Programa de Pós-Graduação em Biotecnologia, Universidade Católica Dom Bosco, Campo Grande 79117-900, Brazil; marlonhenrique6@gmail.com (M.H.C.); biatmeneguetti@gmail.com (B.T.M.); ocostab@gmail.com (B.O.C.); dfbuccini@gmail.com (D.F.B.); oshiro.kgn@gmail.com (K.G.N.O.); dyrosha@gmail.com (S.L.E.P.); rf7085@ucdb.br (C.M.E.C.); ludovico.migliolo@gmail.com (L.M.); 2 Centro de Análises Proteômicas e Bioquímicas, Pós-Graduação em Ciências Genômicas e Biotecnologia, Universidade Católica de Brasília, Brasília 70790-160, Brazil; 3 Programa de Pós-Graduação em Patologia Molecular, Faculdade de Medicina, Universidade de Brasília, Brasília 70910-900, Brazil; 4 Programa de Pós-Graduação em Bioquímica, Universidade Federal do Rio Grande do Norte, Natal 59078-900, Brazil

**Keywords:** antimicrobial peptides, non-lytic peptides, bacterial membranes

## Abstract

The advent of multidrug resistance among pathogenic bacteria has attracted great attention worldwide. As a response to this growing challenge, diverse studies have focused on the development of novel anti-infective therapies, including antimicrobial peptides (AMPs). The biological properties of this class of antimicrobials have been thoroughly investigated, and membranolytic activities are the most reported mechanisms by which AMPs kill bacteria. Nevertheless, an increasing number of works have pointed to a different direction, in which AMPs are seen to be capable of displaying non-lytic modes of action by internalizing bacterial cells. In this context, this review focused on the description of the in vitro and in vivo antibacterial and antibiofilm activities of non-lytic AMPs, including indolicidin, buforin II PR-39, bactenecins, apidaecin, and drosocin, also shedding light on how AMPs interact with and further translocate through bacterial membranes to act on intracellular targets, including DNA, RNA, cell wall and protein synthesis.

## 1. Introduction

The World Health Organization (WHO) has identified antimicrobial resistance as one of the three major threats to human health [1]. Bacteria can be efficient in the synthesis and sharing of genes involved in the development of antibiotic resistance mechanisms, leading to negative outcomes in the clinic [2]. This inefficiency may be related to the intrinsic resistance of a bacterium to a specific antibiotic, which can be explained by its ability to resist the action of this drug as a result of inherent structural or functional characteristics [3]. Therefore, the dissemination of antibiotic resistance factors, along with the misuse of these drugs, has made drug design a broad field of research [4]. In this scenario, the antimicrobial peptides (AMPs) have been considered as an alternative to conventional antibacterial treatments [5].

AMPs can be produced as part of the host’s defense system (innate immune system) during an infection process [6]. These peptides belong to a broad group of molecules produced by many tissues and cell types in a variety of organisms, including plants, invertebrates, vertebrates, fungi, and bacteria [7]. The majority of AMPs are composed of relatively small (<10 kDa), cationic and amphipathic molecules, mostly consisting of 6 to 50 amino acid residues [8]. Moreover, AMPs have often been reported for their diverse biological activities, more specifically, antibacterial activities [9]. The different amino acid compositions lead to structural properties in terms of amphipathicity, net positive charge, shape and size, which favor interaction with microbial surfaces, insertion into lipid bilayers and induction of membrane damage [10]. It is proposed that AMPs firstly bind to biological membranes and then, due to their amphipathic arrangement, insert into the bilayer by breaking the lipid chain interactions [11]. The mechanisms of action associated with destabilization and disruption of bacterial membranes have been widely described, triggering mechanisms known as the carpet model, toroidal pore, barrel type, detergent, and several other variations [12,13,14]. In addition to AMPs’ membrane disruptive properties, studies have suggested that these peptides may also affect bacterial viability by acting via non-lytic pathways [15].

Diverse works assume that AMPs may present intracellular targets [15]. However, the mechanisms by which some AMPs are capable of penetrating bacterial cells are still under investigation [16]. It has been suggested that some peptides (e.g., proline-rich AMPs) can bind to the bacterial surface followed by their translocation into the cell through the formation of transient pores and, finally, acting on intracellular targets [17,18]. Additionally, works have proposed that AMPs can translocate through the membrane without forming pores, which may include receptor-mediated processes [19]. Once these molecules cross the bacterial membranes, they may target intracellular macromolecules and bioprocesses, including DNA replication and transcription inhibition [20,21]. Additionally, AMPs have been proved to inactivate bacterial chaperones involved in protein folding, also leading to bactericidal effects by inhibiting the synthesis of proteins [18,22].

In this context, some advantages have been attributed to non-lytic AMPs in terms of clinical applications. From the therapeutic point of view, AMPs may present great specificity with their intracellular target, which may hinder the development of resistance mechanisms. Moreover, this specificity for intracellular bacterial targets could also lead to reduced toxicity toward healthy human cells [15]. Therefore, this review will focus on the main non-lytic AMPs described to date, including indolicidin, buforin II, PR-39, bac7, apidaecin, and drosocin. Thus, although previous review articles have extensively described AMP intracellular mechanisms of action, here we provide an all-in-one overview of how non-lytic AMPs first interact with and further translocate across bacterial membranes to act on intracellular bacterial components, finally leading to cell death. We also provide a detailed description of the antibacterial, antibiofilm and anti-infective properties of these peptides in vitro and in vivo. Taken together, the data here summarized may provide useful information on the most promising non-lytic AMPs, and how these peptides could be used as model molecules for drug design strategies aiming at antibacterial therapies.

## 2. Indolicidin—A Tryptophan/Proline-Rich Peptide

The first indolicidin was isolated from cytoplasmic granules of bovine neutrophils and, at that time, it was considered the shortest peptide discovered [23]. Indolicidin is a tryptophan/proline-rich AMP belonging to the cathelicidin family and constituted of 13 amino acid residues [23] that has shown antibacterial properties against Gram-positive and -negative bacteria [24]. In terms of structural profile, indolicidin is dynamic, as in an aqueous solution it is unstructured [25], but it adopts a poly-L-proline type II helix or extended structures (Table 1) in membrane-like conditions [26,27].

### 2.1. Indolicidin Interacts with and Translocates through Bacterial Membranes

**Figure 1:**
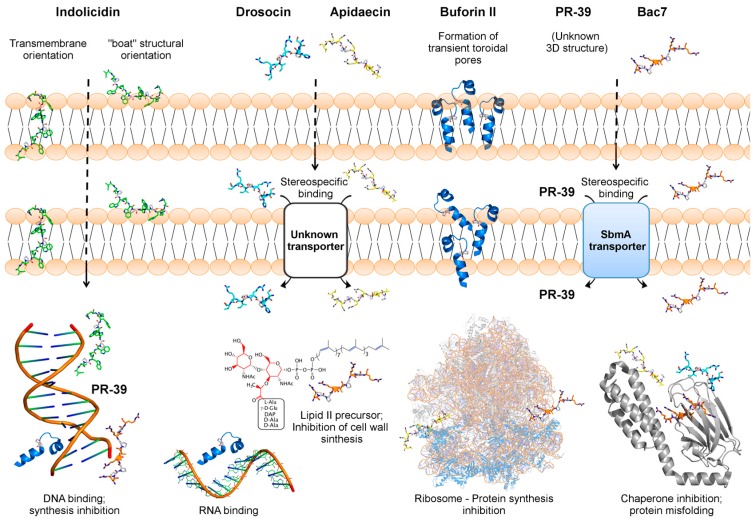
Representation of the membrane translocation mechanisms and intracellular targets for indolicidin (green sticks—PDB 1g8c), PR-39 (name only), bac7 (orange sticks—PDB: 5f8k), apidaecin (yellow sticks—PDB: 5o2r), drosocin (cyan sticks—PDB: 4ezr) and buforin II (blue—PDB: 4kha). Indolicidin adopts a “boat-type” arrangement or transmembrane orientations to cross both the outer membrane (OM) and inner membrane (IM) to bind DNA, whereas buforin II forms transient toroidal pores, thus internalizing the bacterial cell to target DNA and RNA. Apidaecin and drosocin require an IM transporter (e.g., membrane permease) to reach the bacterial cytosol and target chaperones and ribosomes. Similarly, bac7 and PR-39 require an SbmA transporter to cross the IM and then interact with DNA, chaperones and lipid II precursors (the later is exclusive to bac7). Proline residues are highlighted as white sticks in all peptides. The tridimensional structure of buforin II is not deposited in the Protein Data Bank. Therefore, buforin II structure was extracted from the N-terminus region of the histone H2A (from which this peptide is derived), for representation purposes.

Structural studies of indolicidin in contact with lipid bilayers started in the 1990s. At first, it was proposed that indolicidin adopted a poly-L-proline type II helix upon interaction with 1-pamitoyl-2-oleoyl-sn-glycero-3-phosphocholine(POPC)/1-palmitoyl-2-oleoyl-sn-glycero-3-phosphoglycerol (POPG) liposomes (7:3 lipid-to-lipid ratio), which was further correlated to indolicidin’s ability to bind lipopolysaccharides and cross the *Escherichia coli* outer membrane by self-promoted uptake [25]. Years later, this poly-l-proline type II helix structural profile was reviewed, opening a new perspective on indolicidin’s structure by the formation of extended and β-turn structures [27]. Nuclear magnetic resonance (NMR) studies with indolicidin have enabled researchers to clarify this controversy regarding indolicidin’s structure in membrane-like conditions, including zwitterionic dodecylphosphocholine (DPC) and anionic sodium dodecyl sulfate (SDS) micelles. NMR spectra of indolicidin in these conditions have shown that this peptide adopts an extended conformation from residues 3 to 11, with two half-turns at residues 5 and 8 when in contact with DPC [28]. A similar extended conformation was observed in SDS from residues 5 to 11. However, it lacks the bend in the C-terminal region. Further investigations revealed that, in contact with DPC, indolicidin’s conformation seems to be ideal for its intercalation between the DPC molecules. Moreover, based on hydrogen bond pattern, peptide–lipid charge distribution and membrane location, two main membrane insertion modes have been proposed, including the direct penetration into one of the bilayer leaflets via a “boat” structural orientation, and a transmembrane orientation (Table 1 and Figure 1) [28]. In addition to indolicidin–micelle interactions, evidence of multiple structural profiles involved in membrane binding has also been reported in aqueous solution and 50% 2,2,2-trifluoroethanol (TFE) in water [29]. Therefore, it has been suggested that such structural plasticity seems to be related to different combinations of contact between the two WPW motifs in indolicidin’s sequence [29].

The trajectory of indolicidin has also been investigated through molecular dynamics (MD) in membrane environments. Hsu and Yip [30] developed a study with indolicidin, in which the simulations were performed on single lipid bilayers of dioleoylphosphatidylcholine (DOPC), distearoylphosphatidylcholine (DSPC), dioleoylphosphatidylglycerol (DOPG), and distearoylphosphatidylglycerol (DSPG) for 50 ns. The results indicated that indolicidin was partitioned between water and bilayer for all systems. The results suggest that electrostatic interactions are important in the initial attraction of the peptide/membrane. This approach was faster with the anionic lipids (DOPG and DSPG) and there was hydrogen bonding between the peptide side chains and the phospholipid head groups in all simulations. Intermolecular hydrogen bonds were formed between the tryptophan residues from indolicidin, indicating that it is not only by electrostatic interactions that the association with anionic membranes occurs. The authors also observed a decrease in membrane thickness caused by this peptide, along with interdigitation of lipid tails. However, intermolecular hydrogen bonds were not observed when simulating indolicidin in contact with zwitterionic DOPC and DSPC membranes.

More recently, Tsai et al. [31] performed a work with a synthetic analogue of indolicidin, called SAP10, which preserved the non-lytic behavior of the parent peptide but reduced its cytotoxicity against mouse fibroblasts (NIH/3T3). MD simulations of these two peptides (parent and analogue) were performed in the presence of POPC lipid bilayers and the results compared with small-angle X-ray scattering (SAXS). Carbon-carbon order parameters of the lipid acyl chains were used to measure the perturbation in the membrane. For indolicidin, there was a decrease in the values of lipid acyl chains when compared to the isolated membrane, whereas for SAP10, the values did not change significantly [31]. This indicates that both molecules interact with the membrane. However, the indolicidin disturbance is more evident than the SAP10 peptide. The authors associated this lower perturbation with the fact that SAP10 has fewer tryptophan residues, an amino acid that is usually associated with peptide stability in membranes [31].

### 2.2. Indolicidin Antibacterial Properties

Diverse studies have focused on the biological characterization of indolicidin. In the first studies conducted with this AMP, indolicidin showed antibacterial activity against *E. coli, Pseudomonas aeruginosa, Staphylococcus aureus*, *Staphylococcus epidermidis,* and *Salmonella typhimurium* (Table 2) [25,51]. These activities have been directly correlated to the large number of tryptophan and proline residues in indolicidin’s sequence [51]. Nonetheless, the presence of these residues has also been related to the hemolytic activity of this peptide, thus representing an obstacle for its application in clinical trials [52].

Considering that hemolytic and cytotoxic effects represent a bottleneck in taking AMPs into the clinic, indolicidin analogues have been developed. Over the years, different strategies have been carried out to enhance the therapeutic potential of this peptide (Table 1) and, during these investigations, important findings were reported. In a study by Ryge et al. [53], indolicidin analogues were developed based on the sequence ILPXKXPXXPXRR-NH_2_, where tryptophan (labeled with X) residues were altered by an N-substituted class of non-proteogenic residues or by glycine. A total of 28 indolicidin analogues were developed, out of which 22 presented improved antibacterial properties against *S. aureus* and *E. coli* (Table 2). In that same work, non-natural constructs were further submitted to modifications to boost the antibacterial activity of the analogue peptides. For this, phenylalanine residues were added at positions 4, 6, 8, 9, and 11 [53]. As a result, the authors observed that tryptophan might not be essential to maintain the antibacterial activity of the parent indolicidin, as the phenylalanine-containing analogues presented higher minimal inhibitory concentration (MIC) values against *E. coli* and *S. aureus*, as well as lower hemolytic activities.

Amide bond modifications have also been performed aiming at generating analogues with greater stability and antibacterial activity [54,55]. Kim et al. [56], for instance, altered the amide bonds of indolicidin by reduced amide bonds ψ[CH_2_ NH] in the pseudopeptide analogues, called ID, ID-I, ID-W and ID-IW (Table 2). Among them, the pseudopeptide (ID-IW) containing two reduced amide bonds not only presented reduced hemolytic effects, but also improved resistance to enzymatic degradation [56]. Moreover, the antibacterial potential of the parent peptide (indolicidin) was preserved in ID-IW, which was capable of inhibiting *Bacillus subtilis*, *Micrococcus luteus*, *S. aureus* and *E. coli* strains.

More recently, indolicidin has also been used for the generation of hybrid AMPs. In a study by Jindal et al. [57], 13 hybrid peptides were developed based on two AMPs, indolicidin and ranalexin, which is derived from bullfrog (*Rana catesbeiana*) skin [58]. Among them, five analogues (RN7-IN10, RN7-IN9, RN7-IN8, RN7-IN7 and RN7-IN6) presented antibacterial activity against 30 clinical isolates from the genus *Pneumococcus* (Table 2). These authors also used the analogues RN7-IN10 and RN7-IN8 (lower cytotoxicity) to treat mice infected with *Streptococcus pneumoniae*. It was observed that, at the concentration of 5 mg·kg^−1^, the peptides showed no activity. On the other hand, 10% of the animals survived after treatment with RN7-IN10, at 10 mg·kg^−1^, whereas a 30% survival rate was observed for those animals treated with RN7-IN8 at the same concentration. Finally, the highest survival rates of 30% and 50% were reported for the groups treated with 20 mg·kg^−1^ of RN7-IN10 and RN7-IN8 [57]. Interestingly, it was also shown that RN7-IN10 and RN7-IN8 synergize (Table 1), as animals treated with 10 mg·kg^−1^ of each peptide in combination presented a survival rate of 60%. Among all the tests performed, RN7-IN8 presented the most promising activities, besides being highly effective in the treatment of bacteremia [57].

Apart from the antibacterial activity of indolicidin against bacteria in their planktonic mode of growth, studies have also evaluated this AMP on bacterial biofilms. However, in contrast to the promising antibacterial effects of indolicidin and its analogues, antibiofilm studies have shown that the mechanisms by which this AMP acts are not effective on biofilms. Pompilio et al. [59] analyzed the antibiofilm activity of indolicidin against clinical isolates of *P. aeruginosa*, *Stenotrophomonas maltophilia,* and *S. aureus*, but no activity was observed at the maximal concentration tested. In a study by Dosler et al. [60], indolicidin was tested against *S. aureus* and methicillin-resistant *Staphylococcus aureus* (MRSA) biofilms. Despite presenting low MICs, antibiofilm properties were reported only at 40-fold higher concentrations. Overall, these data reinforce the theory that antibacterial and antibiofilm properties in AMPs are most likely to be independent. 

### 2.3. Indolicidin Targets Bacterial DNA

Some AMPs are capable of directly interacting with DNA and/or RNA, thus interfering with their synthesis, replication and translation processes [80,81]. Indolicidin, at high concentrations, increases the permeability of the bacterial cell and, consequently, reaches the cytosol to inhibit, exclusively, DNA biosynthesis (Table 1 and Figure 1) [82]. Hsu et al. [29] performed gel retardation and fluorescence studies to confirm that indolicidin binds to DNA. Besides, different single- and duplex-strand DNAs were immobilized on a biosensor surface and the association/dissociation of indolicidin was monitored. It was demonstrated that indolicidin bound strongly to ds-[AT], ds-[CG] and ds-[AG], but only weakly to ds-[GT]. The authors further suggested that indolicidin’s amphipathicity plays a crucial role in its ability to bind to nucleic acid and, thereby, kill bacteria. Moreover, the data reported by those authors suggest that indolicidin’s mechanism of action involves an initial stage of electrostatic binding to the DNA duplex phosphate groups, followed by its insertion into the DNA groove [29]. More recently, the structural and mechanistic features that favor indolicidin’s DNA-binding property were investigated through the combination of spectroscopy and microscopy methods [32]. It has been shown that the central PWWP motif plays a key role in the indolicidin/duplex DNA stabilization, as mutations in the central WW pair significantly impaired indolicidin’s DNA-binding activity [32].

## 3. Buforin II—A Frog-Derived Peptide that Internalizes Bacterial Cells

Buforin has been described as an effective non-lytic AMP family. The buforin family comprises AMPs that have complete sequence identity with the N-terminal region of the histone H2A, which interacts directly with nucleic acids [83]. Among buforins, buforin II has attracted particular interest due to its broad-spectrum activities against microorganisms when compared to other α-helical AMPs [61]. This peptide was obtained by treating buforin I, which is derived from the stomach tissue of the Asian toad *Bufo bufo gargarizans*, with an endoproteinase Lys-C, thus resulting in the generation of a 21 amino acid residue peptide (TRSSRAGLQFPVGRVHRLLRK), named buforin II [61]. Buforin II has a helical-helix-propeller structure (Table 1), which is amphipathic in hydrophobic environments. In addition, the suggested mechanisms of action of this peptide against bacteria include DNA- and RNA-binding properties after translocation across the lipid bilayer, without causing cell lysis (Figure 1) [84,85].

### 3.1. Buforin II Translocates Membranes by the Formation Of Transient Toroidal Pores

The first NMR structural study performed with buforin II revealed a coil-to-helix transition when this peptide is transferred from hydrophilic (water) to hydrophobic (TFE/water mixtures) conditions [33]. Although buforin II is a non-proline-rich AMP, it presents a proline residue at position 11 in its sequence that acts as a helix breaker. Therefore, the amphipathic structure of buforin II consists of a random coil region from Thr^1^ to Ser^4^, followed by a distorted helical structure from Arg^5^ to Phe^10^ and a well-defined α-helix from Val^12^ to Lys^21^ after a hinge at Pro^11^ [33,34]. The presence of a proline hinge in buforin II has been reported as a crucial factor for its cell-penetrating ability. Interestingly, although the proline acts as a translocation promoter in buforin II, its cis-trans isomerization does not affect the translocation mechanism [35]. Confocal microscopy studies have shown that, by performing a single amino acid substitution for proline in buforin II sequence, this peptide’s mechanism of action on bacteria changes from intracellular to membrane active [34]. Similar findings were observed through the investigation of buforin II in contact with membrane bilayers [36]. Compared to magainin II, buforin II translocates more efficiently across lipid bilayers, without inducing lipid flip-flop, suggesting non-membranolytic mechanisms [36].

Additional studies with lipid bilayers have also demonstrated that buforin II causes a positive curvature on membranes [35]. As mentioned above, Pro^11^ distorts the helical segment in buforin II at the N-terminal region, leading to the concentration of basic residues in a limited amphipathic region, which destabilizes pore formation due to peptide–peptide electrostatic repulsions [35]. Therefore, it is proposed that buforin II translocates membranes by the formation of transient toroidal pores with extremely short lifetime to act on intracellular targets (Table 1 and Figure 1). These findings have also been observed in computational studies [84].

### 3.2. Buforin II as a Promising Scaffold for Antibacterial Therapies

The first study to evaluate the antimicrobial activity of buforin II was developed by Park et al. [61], who determined the MICs against diverse Gram-positive and -negative bacteria, and fungi. In addition, that study also revealed that, compared to the AMP magainin II, buforin II was approximately 10-fold more potent against a wide range of microorganisms [61].

Moreover, in a direct comparison with the model AMP magainin II, buforin II has been evaluated regarding its membrane permeabilization, and its hemolytic and antibacterial properties [36]. In this context, studies have shown that buforin II is more efficient at translocating through lipid bilayers than magainin II [36]. Regarding their antibacterial activity against *E. coli*, buforin II exhibited significantly greater activity than magainin II [36]. Interestingly, however, despite their different modes of action on bacteria, both buforin II and magainin II were not hemolytic at concentrations 25-fold higher than their MICs [36].

Over the years, an increasing number of pharmacologic strategies have been applied to AMPs, including their administration in combination with conventional antibiotics (Table 1) [62]. In this context, Cirioni et al. [63] investigated both in vitro and in vivo the antibacterial activity of buforin II (Table 2) and the antibiotic rifampicin (alone and in combination) against *A. baumannii* strains. As a result, in vitro experiments with buforin II showed higher antibacterial activity when compared to rifampicin against susceptible and multidrug-resistant *A. baumannii*. Moreover, the combination of these two antimicrobial agents resulted in a synergistic effect (fractionary inhibitory concentration (FIC) index of 0.312) [63]. In vivo assays were carried out using a model of sepsis in rats, in which the animals were infected with susceptible and multidrug-resistant *A. baumannii*. The groups treated with buforin II had a lower percentage of lethality (40% and 46.6%, respectively) when compared to the control groups (100% and 100%, respectively) and treated with antibiotic rifampicin (93.3% and 93.3%, respectively) [63]. In addition, the treatment with this peptide also reduced bacterial endotoxin and plasma cytokine concentrations when compared to the other groups [63]. As observed in vitro, the combinatory therapy buforin II and rifampicin was more promising (20% lethality rate for susceptible and resistant *A. baumannii*) compared to the results obtained by the treatment with these antimicrobial agents alone. This combination was also reflected in a significant reduction in the concentrations of bacterial endotoxin and plasma cytokines [63]. Therefore, these results demonstrate that buforin II combined with rifampicin has superior efficacy to monotherapy (Table 1).

In another study, Zhou et al. [64] investigated the interaction of buforin II with the conventional antibiotics ranalexin, amoxicillin-clavulanate, ceftriaxone, meropenem, doxycycline, and clarithromycin (Table 1), which are all commonly used in the clinic for the treatment of Gram-positive and -negative bacteria. The combination of buforin II and the above-mentioned antibiotics against 120 clinical isolates was not synergistic, but additive [64]. However, this potent effect of one treatment agent over another still supports the hypothesis that the combination of peptides with antibiotics may represent a promising alternative to antimicrobial monotherapies.

Studies have also been conducted with buforin II analogue sequences (Table 2). For instance, Park et al. [34] have developed buforin II analogues to shed some light on the structural characteristics of buforin II that are crucial for its potent antimicrobial activity. Therefore, a series of N- and C-terminal truncated buforin II fragments or analogues with amino acid substitutions were designed and evaluated for their antimicrobial activity and mechanism of action [34]. As a result, the analogues BUF (5–21—N-terminal truncation), BUF (5–13—N-terminal truncation) with three repeats of the C-terminal regular RLLR motif, named BUF (5–13)-[RLLR]_3_), were more potent against bacteria than their parent peptide, buforin II (Table 2). In contrast, additional N-terminal truncation, or removal of four amino acids from the C-terminal of buforin II, resulted in analogues with progressive decrease or null antimicrobial activity [34]. These results demonstrate the importance of the C-terminal helical region (residues 18 to 21) in buforin II antimicrobial activity, whereas the N-terminal random coil region seems not to play a key role [34]. Therefore, the systematic study of the structure-activity relationship of buforin II and its analogues has shown that the effectiveness of cell penetration in terms of antimicrobial potency depends on the α-helical content of this AMP [34].

Based on the findings cited above, Hao et al. [65] designed and synthesized a novel, 21-amino acid residue buforin II analogue, called BF2-C. This analogue is constituted by the N-terminal residues 5–13 from buforin II, in addition to three repeats of the C-terminal α-helical motif (RLLR) from this same peptide. Moreover, BF2-C also presents a single substitution, in which a valine residue was replaced by a leucine residue at position 12 in the parent peptide buforin II [65]. These modifications resulted in increased hydrophobicity of the amphipathic α-helix at the C-terminal region of BF2-C. It was observed that BF2-C showed remarkable antimicrobial activities against Gram-positive and -negative bacteria (Table 2), compared to its parent peptide [65]. These results suggest that the α-helical content in buforin-like peptides may be directly correlated with their increased antibacterial potential. Furthermore, structure-activity ratio analyses revealed that cell penetration efficacy and DNA affinity were critical factors in determining the antimicrobial potency of BF2-C. Therefore, these results provide important information on the development of novel potent peptide-based drugs that act intracellularly [65].

Strategies of amino acid replacement have also been applied for the generation of buforin II analogues. Jang et al. [66] designed four analogues, named Buf-IIIa to Buf-IIId, based on the buforin IIb (BUF2-B) respecting the following criteria: (i) the non-alteration of the structural characteristics important for the antimicrobial activity of buforin IIb, and (ii) the conservation of global hydrophobicity, which provides the effective antimicrobial activity of AMPs (Table 2). In that study, all Buf-III analogues had similar structures and mechanisms of action to buforin IIb. Regarding their antimicrobial activity against the tested pathogens (bacteria and fungi), Buf-IIIb and Buf-IIIc presented ≥2-fold higher antibacterial and antifungal activities compared to the parent peptide. Moreover, the hemolytic activity against human erythrocytes was decreased in those analogues, resulting in a 7-fold improvement in their therapeutic index (62.5 for buforin IIb and 444 for Buf-IIIb and IIIc). Therefore, these results suggest that Buf-III analogues may be promising candidates to complement conventional antimicrobial therapy [66].

A buforin II analogue (BF2-A) has also been evaluated in an alternative drug design approach (Table 1), involving its conjugation with a peptide nucleic acid (PNA) to inactivate *E. coli* strains [67]. Due to BF2-A’s intracellular mechanism of action, this peptide would be an efficient vehicle for the release of PNA within the bacterial cells, which in turn targets the *acpP* gene. This gene is essential in fatty acid biosynthesis and, therefore, its regulation interferes with the cell wall organization. Thus, the antimicrobial activity observed against *E. coli* treated with BF2-A and PNA were successfully achieved by the silencing of the target gene promoted by the conjugate [67].

### 3.3. Buforin II Targets DNA and RNA

Apart from indolicidin, the AMP buforin II binds to DNA after its translocation through *E. coli* membranes [34]. The proposed model for buforin II is the formation of a transient toroidal pore (Table 1 and Figure 1), similar to magainin II. The lifetime of the pore is shorter and, as a consequence, the translocation rate is increased due to the disintegration of the pores [35,36]. Once in the cytosol, buforin II binds to DNA and RNA (Figure 1), as shown by Park et al. [85]. The strong affinity of this peptide for nucleic acids has been shown to depend highly on the complementarity between the sequences of buforin II and the N-terminal region of the H2A histone [83].

## 4. PR-39 and bac7—Two Proline/Arginine-Rich Peptides

Proline/arginine-rich peptides have been described and characterized by the presence of a repeating PRPR motif [17]. Arginine and proline residues can facilitate access to the intracellular region of the target bacteria to effectively inactivate these pathogens [17]. The proline/arginine AMP, named PR-39, was firstly isolated from pigs’ intestines [86]. This peptide is constituted of 39 amino acids, with high contents of proline and arginine residues [87]. The large amount of proline residues gives the PR-39 greater stability for degradation by serine proteases, leading to a longer half-life [68,88]. Over the years, it has been shown that PR-39 acts as an antibacterial and wound healing agent (Table 1) [69]. Moreover, when targeting bacteria, PR-39 acts on DNA and/or protein synthesis (Figure 1) [89].

Similarly, the bactenecin-like peptide, bac7, was firstly isolated from bovine neutrophils, and also constitutes a proline/arginine-rich AMP [90,91]. This peptide has shown antibacterial potential toward *E. coli*, *Klebsiella* sp. [90] and may also be effective against *S. epidermidis* [92]. Moreover, the mechanisms by which bac7 exerts its antibacterial properties have been elucidated, and involve a DnaK-binding mode of action (Figure 1) [93].

### 4.1. PR-39 and bac7 Membrane Translocation Require an Inner Membrane Transporter

The bacterial inner membrane (IM) transporter, SbmA, is required for bac7 and PR-39 cellular uptake (Table 1, Figure 1). This IM protein is constituted of 406 amino acid residues with seven or eight transmembrane-spanning domains that facilitate the internalization of glycopeptides, AMPs and PNA oligomers into Gram-negative bacterial cells [40]. To investigate and confirm the role of SbmA in bac7(1–35) (a bac7 truncated fragment) and PR-39 internalization in bacteria, studies have shown that *E. coli* carrying a point mutation in the *sbmA* gene, along with other *sbmA*-null mutants, are resistant to the administration of these two AMPs [37]. These findings have been further confirmed by fluorescence analyses, in which fluorescently labeled bac7(1–35) revealed lower cell internalization properties in *sbmA* mutated *E. coli* [37]. More recently, the functional characterization of SbmA in the presence of bac7(1–35) was carried out [40]. In that work, it was proposed that bac7(1–35) uptake is not ATP-dependent, but requires the presence of a transmembrane electrochemical gradient [40]. Moreover, it was found that bac7(1–35) directly binds to SbmA with high affinity, finally leading to conformational changes in this transporter [40].

### 4.2. PR-39 Antibacterial Properties

One of the first studies conducted with PR-39 demonstrated that this AMP inhibits *E. coli*, *S. typhimurium* and *Salmonella choleraesuis* growth (Table 2) [69]. In addition, this AMP also causes bacterial death, with the highest activities reported against *E. coli* [69]. Similar findings were observed by Jeon et al. [94], who considered the antibacterial potential of PR-39 similar to those obtained for ampicillin and gentamicin.

As for indolicidin, PR-39 analogues have also been generated aiming at an optimized therapeutic index. Studies have reported the evaluation of PR-39 truncated analogues, including PR-39 (1–26), PR-39 (1–22), PR-39 (1–18), PR-39 (1–15), PR-39 (16–39), PR-39 (20–39) and PR-39 (24–39), against different bacterial strains (Table 2). As a result, the most effective analogues were PR-39 (1–26), PR-39 (1–22), PR-39 (1–18) and PR-39 (1–15), presenting similar minimal bactericidal concentrations of PR-39 against *E. coli* and *Bacillus globigii* [68]. These findings suggest that shorter N-terminal fragments from the parent PR-39 could be developed aiming at conserved/improved antibacterial properties, allied to a lower cost of synthesis.

In terms of in vivo antibacterial properties, PR-39 has been used for the treatment of sepsis in mice through endotoxin neutralization. Studies have shown that PR-39, when administrated with lipopolysaccharides (LPS), leads to a decreased release of nitric oxide (NO) by mice cells, thus reducing the cellular stress and, consequently, improving the survival rates of the treated animals in a sepsis model (Table 1) [70].

### 4.3. Bac7 Antibacterial Properties

Bac7 and its truncated analogues have been tested against numerous Gram-negative bacteria, including *E. coli*, *A. baumannii*, *K. pneumoniae* and *Salmonella enterica*, revealing the highest inhibitory potential for bac7(1–35) (Table 2) [95]. Moreover, the antibiofilm activity of bac7 has also been investigated against clinical isolates of *S. maltophilia* and *S. aureus* and *P. aeruginosa*. However, as for indolicidin, promising results were not obtained at the maximal concentration tested [59]. On the other hand, an in vivo study demonstrated that treatment with bac7 protects rats against *E. coli* endotoxins, thus avoiding septic shock (Table 1) [71].

The antibacterial activity of bac7(1–35) has also been evaluated in vivo using a murine model of *Salmonella* infection, resembling systemic infections in humans [96]. Therefore, it has been observed that untreated animals (control) survived for 10 days post-infection, whereas those animals treated with bac7 (75 mg·kg^−1^) survived for 24.5 days post-infection. In addition, 35% of the animals treated with bac7 recovered completely from the infection, thus significantly reducing the mortality rates [96]. Years later, Benincasa et al. [96] used a 20 kDa polyethyleneglycol (PEG) to improve the effectiveness of bac7 against *Salmonella* infection in mice models. After intraperitoneal administration, the animals were observed for 24 h. Greater activity of bac7 and PEG were observed, although it was found in organs (e.g., kidneys and liver) for longer periods. Therefore, the association of PEG with bac7 proved to be a promising modification for the therapeutic applicability of this AMP (Table 1) [97].

### 4.4. PR-39 and bac7 Target Bacterial Protein Synthesis

One of the mechanisms by which non-lytic AMPs lead bacteria to death is the inhibition of protein synthesis. The proline/arginine-rich AMP PR-39 is known to rapidly cross bacterial cell membranes, without causing significant damage. Once in the intracellular compartment, this AMP inhibits proteins involved in DNA replication (Table 1 and Figure 1). The mechanism of action is attributed to PR-39′s proteolytic activity, which causes the degradation of proteins associated with DNA replication, leading to the secondary inhibition of DNA synthesis [72]. In an attempt to find out the exact mechanism by which PR-39 exerts its antibacterial properties, Ho et al. [73] carried out a proteome microarray study with *E. coli* to systematically identify the intracellular protein targeted by this AMP. The inhibitory effects of PR-39 on diverse metabolic pathways have been confirmed, including those for translation, transport and metabolism of nucleotides, transport, and metabolism of coenzymes and others [73].

Protein and RNA synthesis have also been targeted by the non-lytic AMP bac7 (Table 1 and Figure 1) [38,90]. Mardirossian et al. [39] showed that bac7 (1–35) blocks protein synthesis by targeting ribosomal proteins. Moreover, the authors also proposed that this mechanism could prevent additional co-linear events, including the interaction of cotranslational chaperones with ribosomes, which is a known mechanism to ensure the translation of any polypeptide chain [39]. More recently, this mechanism was further explored, revealing that bac7(1–35) blocks the peptide exit tunnel in 70S ribosomes from *Thermus thermophiles* [98]. It was also concluded that this mechanism occurs through the interaction of bac7(1–35) with antibiotic-binding sites, thus interfering with the initial step of translation [98]. In addition, it has been proposed that bactenecins also target cell wall synthesis by binding to the lipid II precursor (Table 1 and Figure 1) [41]. These data support the idea that a single AMP may have multiple mechanisms of action simultaneously, which contributes to the lower occurrence of bacterial resistance to AMPs.

Although bac7(1–35) has been widely described as a non-lytic AMP that internalizes bacterial cells through the transporter SbmA, it has been shown that this mechanism varies depending on the characteristics of the target bacterial species. For instance, Runti et al. [42] reported that *P. aeruginosa* (PAO1) cells are inactivated by bac7(1–35) through cellular membrane disruption, which differs from what has been observed against *E. coli*. Interestingly, by expressing the SbmA transporter in *P. aeruginosa* (PAO1) it was found that bac7(1–35) internalization was enhanced, along with higher bacterial resistance to membrane disruption [42]. Therefore, this evidence supports the idea of bac7′s (1–35) multiple mechanisms of action, which are highly dependent on the strain tested.

## 5. Apidaecin and Drosocin—Two Non-Lytic AMPs Derived from Insects

Apidaecin was the first proline-rich AMP isolated from bees in the mid-1980s. Apidaecin comprises an 18–20 amino acid residue peptide with proline and arginine repetitions along its sequence [43]. In contrast, drosocin is a peptide isolated from the fruit fly (*Drosophila melanogaster*), which was first reported by Bulet et al. [44]. Drosocin is composed of 19 amino acid residues in length and shares a high degree of sequence homology with apidaecin [44]. This peptide is characterized by three PRP motif repeats and glycosylation of threonine residues, which is suggested to be intrinsically related to its antibacterial properties [44,99]. Moreover, cytotoxic effects have not been reported for this peptide, reinforcing its therapeutic applicability [99,100].

### 5.1. Apidaecin and Drosocin Depend on Membrane Receptors to Internalize the Target Cell

Initial studies on the structure and membrane translocation of apidaecin peptides have suggested that the antibacterial activities of these peptides are intrinsically related to the presence of PXP motifs, which contribute to the ordered formation of oligomers that facilitates the entry through the bacterial outer membrane (OM) [101]. Nevertheless, although apidaecin’s functional oligomers are capable of translocating across the OM, evidence suggests that its internalization and translocation across the IM are facilitated by specific interaction with membrane permeases and transporters (Table 1 and Figure 1) [78]. Moreover, it seems that such interaction is energy-driven, irreversible and stereospecific (Figure 1), as all-D-apidaecin (apidaecin constituted entirely of d-amino acids) does not bind to periplasmic or IM components [78].

As for apidaecin, drosocin has also been suggested to internalize bacterial cells through interactions with IM receptor/channels [48]. Drosocin is glycosylated at Thr^11^, which has been characterized as a key factor for its antibacterial activities and, therefore, has been investigated in NMR structural studies. In general, spectra recorded in water indicate a high population of random coil arrangements for both glycosylated and non-glycosylated forms, whereas the presence of 50% TFE/water mixtures induces the formation of turns [49]. Although no significant differences were detected for the random coil arrangements in water, the glycosylated and non-glycosylated forms differ greatly in the folded conformations, especially at residues 10 to 13 (extended turn) and 17 to 19 (tightening of the downstream turn) in the glycosylated form. Additional studies have also shown that not only is the glycosylation at Thr^11^ crucial for drosocin’s internalization into bacterial cells, but also its chirality [48]. Similarly to apidaecin, it has been reported that drosocin’s action on bacterial cells is stereospecific, as its D-enantiomers are incapable of internalizing bacterial cells. These findings re-emphasize the receptor-driven mechanism by which drosocin acts (Table 1 and Figure 1). However, although this mechanism has been proposed for both apidaecin and drosocin, the specific target of these non-lytic AMPs on the periplasmic space or IM is still under investigation.

### 5.2. Apidaecin Antibacterial Properties

The first study carried out with apidaecin demonstrated that the activity of this peptide does not depend on cell membrane lysis [45,72]. Years later, when tested against bacteria, apidaecin was proved to cause bacterial cell death without triggering membrane destabilization [78]. It is presumed that the apidaecin C-terminal region (PRPPHPR (L/I)) is responsible for its non-lytic mechanism of action [45,46]. In terms of biological activities, apidaecin has been characterized for its antibacterial effects against numerous Gram-negative bacteria, including *E. coli* [102], *K. pneumoniae* [103] and *P. aeruginosa* (Table 2) [74].

Apidaecin analogues have been developed for improved antibacterial properties (Table 1). Czihal et al. [102], for instance, performed a robust study regarding the comparison between apidaecin and its analogues (api6, api7, api39 and api88) (Table 2). By modifying the C-terminal region through the inversion of an amide in the analogue api6, the authors reported a 32-fold and 4-fold higher antibacterial potential against resistant *E. coli* and *K. pneumoniae* when compared to the parent peptide, apidaecin [102]. In contrast, by acetylating the N-terminal of the analogue api7 greater stability was observed. However, the antibacterial activity of this analogue was abolished. Interestingly, by performing further modification on api7, including the replacement of Gly^1^ by ornithine or lysine, the antibacterial potential of this analogue was reestablished [102]. Similar findings were obtained for the api39 analogue when replacing the Glu^10^ by an arginine, leading to improved inhibitory effects toward bacteria [102]. Finally, the api88 analogue, which presented the highest net positive charge among all analogues, underwent modifications in the N-terminal region, where acetyl amide (CH_3_CONH–) was replaced by *N, N, N′, N′*-tetramethylguanidine (((CH_3_)_2_N)_2_-CNH-). As a result, a remarkable improvement was observed in the antibacterial activity of api88, which revealed low MIC values against the three strains tested and, therefore, was pinpointed as a promising AMP for therapeutic purposes [102].

Additional studies have also shown that replacing the N-terminal glycine of apidaecin by tetramethylguanidino-L-ornithine led to the generation of a structurally stable analogue, named api137, with promising activity against *E. coli* (Table 2) [75]. Moreover, further investigations demonstrated that removing the C-terminal Leu^18^ residue resulted in a substantial loss of antibacterial activity, suggesting the crucial role of the api137 C-terminal region for its antibacterial potential [76]. Structural stability and resistance to enzymatic degradation have also been investigated in apidaecin Ib by substitutions of arginine/leucine residues with peptoid residues (Table 1). Gobbo et al. [104] engineered peptide–peptoid hybrids based on apidaecin Ib and observed that, although presenting higher stability to degradation, the position at which the peptoid residues lie in the apidaecin hybrids impairs their antibacterial activities. The authors reported that modifications at the N-terminal region of apidaecin Ib only slightly reduced the antibacterial property of the hybrids, whereas peptoid residues in the C-terminal region drastically reduced this property [104], once again reinforcing the relevance of a conserved C-terminal for apidaecin peptides’ antibacterial potential.

### 5.3. Drosocin Antibacterial Properties

As described above, the glycosylation of drosocin residues seems to directly interfere with its biological activities against bacteria. In a study by Bikker et al. [100] the glycosylation of Tyr^6^ and Ser^7^ from drosocin was performed. As a result, the antibacterial activities of Tyr^6^ glycosylated and N-terminal β-Ala drosocin analogues against *E. coli*, *Erwinia herbicola* (currently classified as *Pantoea agglomerans*) and several *S. enterica* serovars, namely *S. panama*, *S. infantis*, *S. montevideo*, *S. typhimurium* and *S. enteritidis* (Table 2), were improved compared to the parent non-glycosylated drosocin (Table 2) [100]. More recently, it was shown that the addition of a monosaccharide at Thr^11^ (GKPRPYSPRPT (αGalNAc)SHPRPIRV) led to a remarkable improvement of antibacterial potential against numerous Gram-negative strains compared to non-glycosylated drosocin [50]. Similar findings were reported for a drosocin analogue with the addition of a disaccharide at Thr^11^ [GKPRPYSPRPT (βGal (1 → 3) αGalNAc) SHPRPIRV] (Table 2) [50]. Taken together, these reports highlight the advantages of modulating drosocin’s structure aiming at screening for optimized activities against pathogenic bacteria (Table 1).

### 5.4. Apidaecin and Drosocin Interact with Bacterial Chaperones

As described above, apidaecin translocation across membranes is receptor-mediated and, according to Castle et al. [78], probably has a component of the permease-type carrier system. It has been shown that apidaecin peptides are capable of causing bacterial protein misfolding and aggregation by interacting with bacterial chaperones (Table 1 and Figure 1). Dnak and GroEL are chaperones that aid in the correct folding and assembly of proteins and, consequently, affect many cellular processes including DNA replication, RNA transcription and protein transport. Structural studies involving the molecular complex DnaK/apidaecin have revealed two binding modes, indicating that DnaK is quite unspecific in terms of peptide-binding. Cross-linking and free-cell translation assays have demonstrated that Api88 and Api137 (apidaecin analogues) bind to the 70S ribosome, leading to protein synthesis inhibition (Figure 1) [76]. Apidaecin and drosocin share a high degree of sequence homology, as well as similarities in their antibacterial activity spectra [77]. Therefore, as for apidaecin, drosocin interacts with intracellular proteins, including the heat-shock proteins DnaK and GroEL to inhibit the DnaK ATPase activity and chaperone-assisted protein folding, respectively [79]. Apart from its chaperone-binding property, apidaecin has also been shown to inhibit release factors in bacteria. Matsumoto et al. [105] reported this unusual mechanism through the in vivo target exploration of apidaecin based on acquired resistance induced by gene overexpression (ARGO assay). In that work, recombinant *E. coli* strains overexpressing proteins involved in translation were treated with apidaecin, among which only one clone overexpressing a peptide chain release factor 1 (PrfA) was selected as a positive candidate. PrfA is known to bind to ribosomes to terminate translation processes by recognizing stop codons in mRNA. Therefore, it was proposed that apidaecin probably binds to ribosomes, competitively, thus inhibiting the termination step of translation [105].

## 6. Conclusions and Future Prospects

Here, the antibacterial properties, membrane translocation processes and intracellular mechanisms of action of specific non-lytic AMPs were reviewed. In general, membrane active and non-lytic AMPs present similar physicochemical properties and, therefore, have a high affinity for membrane-like environments. Membrane active AMPs, including magainin, cecropin, and melittin, are known to firstly establish electrostatic interactions with the target bacterial cell, followed by the accumulation of peptides aiming to achieve a critical concentration that favors peptides’ self-association and penetration into the membrane core [47]. From this point on, different modes of action have been described, including barrel-stave/toroidal pores, “carpet”-like mechanism, peptide–lipid aggregation and amyloid models [106]. It has also been shown that synthetic AMPs are capable of delocalizing membrane-bound proteins, leading to bacterial cell envelope stress response [107,108]. In addition, membrane-associated mechanisms not necessarily lead to cell lysis, as observed for lactoferricin and daptomycin, which cause non-lytic membrane depolarization [109,110], and the human α-defensin 6 (HD6), which forms nanonets that interact with membrane proteins to entangle bacteria [111]. Taken together, these membrane-associated mechanisms trigger a series of negative effects on bacterial homeostasis, including disturbance of ion gradient, loss of metabolites, phospholipid flip-flop, membrane depolarization and loss of membrane symmetry [112].

Although AMPs can rapidly display their actions on bacterial membranes, an increasing number of reports have highlighted that bacteria can easily evade membrane-associated mechanisms by adapting the constitution and proportion of phospholipids in their OM and IM [113], as reported for *E. coli* strains resistant to magainin [114]. Therefore, non-lytic AMPs have been pinpointed for their ability to inactivate bacteria by interrupting vital cellular process, instead of membrane destabilization and disruption. Considering the alarming scenario imposed by bacterial infections, the intracellular mechanisms displayed by non-lytic AMPs appear as an advantage over membrane-active AMPs, as those peptides are less likely to induce bacterial resistance. Moreover, a primary non-lytic mechanism (e.g., peptide–protein interactions aiming at compromising bacterial viability) may trigger a secondary mechanism, thus imposing an additional obstacle for bacterial adaptation to non-lytic AMP administration. In terms of bacterial internalization, we emphasized the role of proline residues in all peptides here described, as this residue has been proved to be a membrane translocation promoter and, therefore, is considered a key feature that could be used for future drug design strategies.

Here we summarize the main molecular mechanisms by which non-lytic AMPs translocate across membranes. These mechanisms involve different AMP arrangements (e.g., “boat-like” and transmembrane orientations, for indolicidin) [28] and the formation of transient toroidal pores, which facilitates non-lytic AMPs (e.g., buforin II) in crossing both the bacterial OM and IM to act on intracellular targets [84]. In addition, the stereospecific binding of AMPs to IM transporters (e.g., apidaecin, bac7, and PR-39) has also been highlighted as a strategy by which these peptides reach the bacterial cytosol to exert their functions [37,78]. These mechanisms have also been reported for another non-lytic AMP, called pyrrhocoricin, which is derived from the European firebug *Pyrrhocoris apterus*. As for bac7, apidaecin, and drosocin, pyrrhocoricin binds stereospecifically to an IM target protein and further enters the cytosol to inhibit chaperone-assisted protein folding by interacting with the molecular chaperone DnaK [115]. Similar findings have been reported for oncocin, a proline-rich AMP derived from the milkweed bug, *Oncopeltus fasciatus* [116].

Apart from the chaperone activity of DnaK, the non-lytic AMPs here described are also capable of binding to lipid II precursor, as well as interfering with DNA, RNA and protein synthesis. Although this review focused on eukaryotic-derived AMPs, it is worth noting that bacteriocins (bacteria-derived AMPs) also present intracellular mechanisms of action. Nisin, for instance, represents a bacteriocin derived from *Lactococcus lactis* that inhibits cell wall synthesis by targeting lipid II [117]. Nevertheless, this AMP has also shown membrane-associated mechanisms by the formation of pores and, therefore, is not considered a non-lytic AMP. In contrast, studies have reported that nukacin ISK-1, which is produced by *Staphylococcus warneri*, is also capable of inhibiting cell wall synthesis, but with no membrane-associated properties [118]. Additionally, in terms of peptide–DNA interaction, the bacteriocin microcin B17, originally isolated from *E. coli*, has been shown to inhibit bacterial DNA gyrase, thus interfering with DNA replication [119]. Finally, bacteriocins have also been proved to act as DNase and RNase, as is the case of colicin family members [120,121].

In general, the non-lytic AMPs here presented have demonstrated promising antibacterial effects on both susceptible and resistant strains, whereas reports of antibiofilm activities are scarce and somewhat insubstantial. Although none of them have effectively reached the market, some have been used as lead molecules for the engineering of antimicrobial agents that have achieved advanced clinical trials. Indolicidin, for instance, was used as a model molecule for the design of omiganan, a 12-amino acid residue peptide rich in tryptophan and proline residues. Compared to indolicidin, omiganan N-terminal tryptophan and proline residues were removed, along with the addition of a lysine residue at the C-terminal and a K5R substitution [122]. Omiganan has been submitted to a total of 11 clinical trials as an antimicrobial agent to prevent and treat *Acnes vulgaris*, atopic dermatitis, seborrheic dermatitis, sepsis, fungaemia, among others (please check, DrugBank accession code DB0661). In addition, bactenecin and an innate defense regulator peptide, called IDR1, have been used as parent peptides for the development of a synthetic host defense peptide, IMX942/SGX942 (dusquetide) [123]. This drug candidate has been indicated for oral mucositis and, currently, is in phase III trials (please check, DrugBank accession code DB11879).

Allied to that, an increasing number of studies have highlighted the great therapeutic potential of the other non-lytic AMPs here described. PR-39, for instance, has shown promising anti-sepsis effects on mice, which are related to endotoxin neutralization [70], whereas apidaecin [75] and drosocin [50] have been used for proof-of-concept studies, defining which determinants modulate the generation of improved analogues aiming at antibacterial therapies. Finally, buforin II has shown a wide applicability, as both the parent peptide and its analogues have revealed synergistic effects with conventional antibiotics [63] and have also been proposed as carrier molecules aiming at gene regulation via PNA [67]. Conversely, it is worth mentioning that, in some aspects, non-lytic AMPs still require further attention. The identification of specific binding sites on the target proteins, ribosomes, DNA and RNA would allow the guided design of improved, strain-specific analogue peptides. Moreover, although efforts have been made on optimized non-lytic AMP analogues, their failure to reach the market, in some cases, still relies on poor in vivo effectiveness, nonspecific cytotoxicity, and bioavailability.

Overall, the data here summarized indicate the biotechnology and pharmaceutical potential of non-lytic AMPs as promising drug leads. However, it also reveals the need for deeper investigations aiming at generating candidates that could be successfully translated to the clinic.

## Figures and Tables

**Table 1:** Summary of the non-lytic AMPs here described in terms of antibacterial potential and applicability, the design strategies for the generation of improved analogues, structural profiles, modes of translocation across bacterial membrane and known intracellular targets.

Non-Lytic AMPs	Antibacterial Potential	Treatment Strategies	Design Strategies	Structural Profile	Membrane Translocation	Intracellular Target	References
**Indolicidin**	Bacteriostatic; bactericide; anti-bacteremia	Monotherapy; synergism between two indolicidin analogues	Amino acid substitution; Amide bond modification; Hybrid peptides	poly-l-proline type II helix; extended structures; β-turn	Transmembrane orientation followed by cell internalization	DNA binding; DNA biosynthesis inhibition	[27,28,29,30,31,32]
Buforin II	Bacteriostatic; bactericide; anti-sepsis	Monotherapy; synergism with rifampicin; additive effects when combined with ranalexin, amoxicillin-clavulanate, ceftriaxone, meropenem, doxycycline, and clarithromycin; conjugation with PNA	Amino acid substitution; truncated analogues	Helical-helix-propeller structure	Formation of transient toroidal pores	DNA and RNA binding	[33,34,35,36]
PR-39	Bacteriostatic; bactericide; anti-sepsis; toxin neutralization; wound healing	Monotherapy	Truncated analogues; amino acid substitution	Extended	Receptor-mediated (SbmA)	Protein and DNA synthesis inhibition	[37,38,39]
Bac7	Bacteriostatic; bactericide; anti-sepsis; immunomodulatory	Monotherapy; synergism; association with PEG	Truncated analogues; amino acid substitution	Extended	Receptor-mediated (SbmA)	Protein and DNA synthesis inhibition; Ribosome; Binding to lipid II precursor; cell wall synthesis	[37,40,41,42,43,44]
Apidaecin	Bacteriostatic; bactericide	Monotherapy	Chemical modifications; amino acid substitution; peptide–peptoid hybrids	Extended	Oligomers formation (OM); Interaction with IM permeases and transporters	DnaK and GroEL, leading to bacterial protein misfolding; Protein synthesis; Ribosome	[45,46,47]
Drosocin	Bacteriostatic; bactericide	Monotherapy	Chemical modifications	Extended	Receptor-mediated (unknown)	DnaK and GroEL, leading to bacterial protein misfolding	[48,49,50]

PEG: polyethyleneglycol; OM: outer membrane; IM: inner membrane; PNA: peptide nucleic acid; SbmA: peptide antibiotic transporter.

**Table 2:** Non-lytic peptides, their source organisms, class, analogue peptides, and antibacterial activity spectrum.

Peptide	Organism	Source	Class	Analogues	Antibacterial Activity Spectrum	MIC Range (μM)	References
Indolicidin	*Bos taurus*	Neutrophils cytoplasmic granules	Tryptophan-rich	N-substituted class of non-proteogenic residues or by glycine; ID, ID-I, ID-W and ID-IW; RN7-IN6 to RN7-IN10	*E. coli, P. aeruginosa, S. aureus, S. epidermidis, S. pneumoniae, M. luteus* and *S. typhimurium*	0.2 to 50	[22,51,53,56,57,58]
Buforin II	*Bufo bufo gargarizans*	Stomach tissue	Helical-helix-propeller peptide	BF2-A; BF2-C; BUF(5–21); BUF(5–13)-[RLLR]_3_; Buf-IIIa to Buf-IIId	*A. baumannii,B. subtilis, C. neoformans, E. coli, L. monocytogene, P. putida, S. aureus, S. dysenteriae, S. hemolyticus, S. marcescens, S. mutans, S. pneumoniae, S. typhimurium* and *Serratia sp.*	0.2 to 3.2	[34,35,36,61,62,63,64,65,66,67]
PR-39	*Sus scrofa*	Porcine neutrophils	Proline/arginine-rich	PR-39 (1–26); PR-39 (1–22); PR-39 (1–18); PR-39 (1–15); PR35	*B. globigii, E. coli, S.typhimurium* and *S.choleraesuis*	1.25 to 20	[68,69,70]
Bac7	*Bos taurus*	Bovine neutrophils	Proline/arginine-rich	Bac7 (1–35); Bac7 (5.35); Bac7 (1–23); Bac7 (5–23); Bac7 (1–16); Bac7 (1–18)	*A. baumannii, E. coli, K. pneumoniae, P. aeruginosa, S. aureus, S. enterica* and *S. maltophilia*	0.06 to 64	[71,72,73]
Apidaecin	*Apis mellifera*	Lymph fluid	Proline/arginine-rich	api6; api7; api39; api88; api137; apidaecin Ib;	*E. coli, K. pneumoniae, P. aeruginosa, S. enteritidis, S. typhimurium*	0.27 to 64	[50,74,75,76,77]
Drosocin	*Drosophila melanogaster*	Abdomen and thoraxes	Proline/arginine-rich	Thr^6^-glycosylated drosocin; β-Ala drosocin; M-drosocin; Di-drosocin	*E. coli, Erwinia herbicola, S. enteritidis, S. infantis, S. montevideo, S. panama, S. typhimurium,* and *M. luteus*	0.25 to 100	[78,79]
